# Prediction of therapeutic intensity level from automatic multiclass segmentation of traumatic brain injury lesions on CT-scans

**DOI:** 10.1038/s41598-023-46945-9

**Published:** 2023-11-17

**Authors:** Clément Brossard, Jules Grèze, Jules-Arnaud de Busschère, Arnaud Attyé, Marion Richard, Florian Dhaussy Tornior, Clément Acquitter, Jean-François Payen, Emmanuel L. Barbier, Pierre Bouzat, Benjamin Lemasson

**Affiliations:** Univ. Grenoble Alpes, Inserm, CHU Grenoble Alpes, Grenoble Institut Neurosciences (GIN), U1216, Eq. “Neuroimagerie Fonctionnelle et Perfusion Cérébrale”, 38700 Grenoble, France

**Keywords:** Medical research, Neurology

## Abstract

The prediction of the therapeutic intensity level (TIL) for severe traumatic brain injury (TBI) patients at the early phase of intensive care unit (ICU) remains challenging. Computed tomography images are still manually quantified and then underexploited. In this study, we develop an artificial intelligence-based tool to segment brain lesions on admission CT-scan and predict TIL within the first week in the ICU. A cohort of 29 head injured patients (87 CT-scans; Dataset1) was used to localize (using a structural atlas), segment (manually or automatically with or without transfer learning) 4 or 7 types of lesions and use these metrics to train classifiers, evaluated with AUC on a nested cross-validation, to predict requirements for TIL sum of 11 points or more during the 8 first days in ICU. The validation of the performances of both segmentation and classification tasks was done with Dice and accuracy scores on a sub-dataset of Dataset1 (internal validation) and an external dataset of 12 TBI patients (12 CT-scans; Dataset2). Automatic 4-class segmentation (without transfer learning) was not able to correctly predict the apparition of a day of extreme TIL (AUC = 60 ± 23%). In contrast, manual quantification of volumes of 7 lesions and their spatial location provided a significantly better prediction power (AUC = 89 ± 17%). Transfer learning significantly improved the automatic 4-class segmentation (DICE scores 0.63 vs 0.34) and trained more efficiently a 7-class convolutional neural network (DICE = 0.64). Both validations showed that segmentations based on transfer learning were able to predict extreme TIL with better or equivalent accuracy (83%) as those made with manual segmentations. Our automatic characterization (volume, type and spatial location) of initial brain lesions observed on CT-scan, publicly available on a dedicated computing platform, could predict requirements for high TIL during the first 8 days after severe TBI. Transfer learning strategies may improve the accuracy of CNN-based segmentation models.

**Trial registrations** Radiomic-TBI cohort; NCT04058379, first posted: 15 august 2019; Radioxy-TC cohort; Health Data Hub index F20220207212747, first posted: 7 February 2022.

## Introduction

Traumatic Brain Injury (TBI) is a leading cause of death and disability in the world. Despite significant progress in their management, half of severe TBI patients will have long-term disabilities. One big issue is to have reliable tools to predict patient outcomes after TBI^1,2^. Models have been developed to predict outcome at 6 months post-trauma such as IMPACT^3^ and CRASH^4^, which include clinical and CT-scan data such as the presence of intracerebral hemorrhagic lesions, midline shift and compression of basal cisterns. However, analysis of CT imaging is qualitative and observer-dependent^5^. Such issue could be solved with the development of artificial intelligence (AI) applied to CT-scan imaging, providing CT-scan quantification^6–11^ or automated delineation of traumatic brain lesions^12,13^.

The BLAST-CT algorithm, developed to automatically delineate intraparenchymal hematoma (IPH), extra-axial hematoma (EAH), intraventricular hemorrhage (IVH) and perilesional oedema (Od) after severe TBI, is to our knowledge the most advanced segmentation tool of TBI lesions on CT-scans^14^.

While predicting TBI patient outcome at 6 months using qualitative analysis of CT scan may be difficult, information contained in initial CT-scan could be used to predict short-term evolution such as the intensity of therapies required for each patient. In severe TBI patients, most therapies are directed to control intracranial pressure (ICP), a strong driver of outcome after severe TBI. Eight ICP-treatment modalities have been then collected to validate a daily scoring system, the TIL sum with a maximum score of 38 points^15^. A TIL sum of 11 points or more is considered as moderate-to-intense requirements for therapies. Although the presence of midline shift and compressed basal cisterns are usually considered as radiological signs of high ICP, nothing is said about the predictive value of CT-scan findings on TIL sum.

We hypothesized that an automated delineation of the most frequent traumatic brain lesions from initial CT-scan could predict a moderate-to-severe TIL sum assessed during the first week after admission to the intensive care unit (ICU).

In this study, we extracted metrics from brain CT-scans representing the volume, the type and the spatial location of injuries using automatic or manual segmentations. Two transfer learning strategies were used to re-train the BLAST-CT algorithm in order to improve the automatic segmentations. Finally, segmentation and classification models were validated using internal and external datasets of patients.

## Methods

### Data retrieval

#### Dataset 1

The first dataset contains 30 head injured patients admitted at the Universitary Hospital of Grenoble (CHUGA) between January 2020 and April 2021 (Radiomic-TBI cohort; NCT04058379). Inclusion was prospective, conditioned to patient agreement and an Abbreviated Injury Score (AIS) ≥ 3^16^, corresponding to the presence of an injury visible in the CT-scan acquired the day of admission. The following clinical data were retrieved at the admission in the Intensive Care unit (ICU): Age, Glasgow Coma Scale (GCS), Mean Arterial Pressure (MAP), presence/absence of antiaggregants and Hemoglobin (Hb) rate. The data needed to compute the TILsum was retrieved daily during the 8 first days in the ICU. Finally, Marshall^17^ and Rotterdam^18^ scores were computed from the CT-scans.

**Figure 1:**
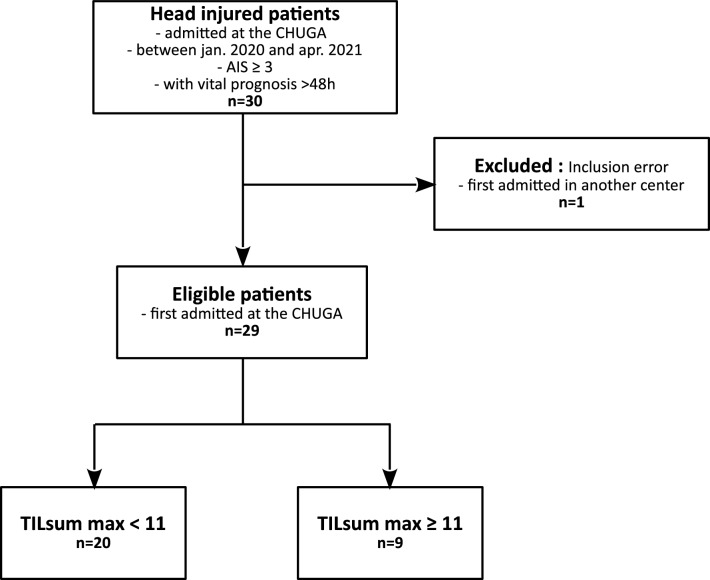
Flowchart of inclusions in Dataset1.

Among the 30 patients of the cohort, one was excluded because of a primary admission outside of the CHUGA, as described on the flowchart on Fig. 1, and 84 CT-scans were finally acquired on these 29 patients. This dataset, characterized in Table 1, was split into a train, validation and test sub-datasets in a 60/20/20 proportion. For obvious independence reasons, all scans of a patient were in the same sub-dataset.

**Table 1:** Characterization of the Radiomic-TBI cohort (mean ± STD [Min–Max]).

	TILsum_Low	TILsum_High
Nb of patients	20	9
Age (years)	48.3 ± 23.2 [18–79]	42.1 ± 18.6 [22–73]
Sex	13M/7F	8M/1F
Weight (kg)	70.8 ± 13.6 [51–100]	76.7 ± 6.1 [68–86]
Height (cm)	175.3 ± 9.8 [160–197]	175.5 ± 5.8 [169–185]
Glasgow Coma Score	9.3 ± 4.3 [3–15]	6.9 ± 4.0 [3–14]
Mean Arterial Pressure (mmHg)	87.8 ± 17.9 [41–115]	92.7 ± 14.6 [67–106]
Hemoglobin (g/l)	125 ± 27.7 [44–155]	129 ± 20.6 [95–164]
Presence Antiaggregants	4/20	0/9
Marshall score	2.6 ± 1.3 [2–6]	4.9 ± 1.1 [3–6]
Rotterdam score	3.0 ± 0.8 [2–5]	4.4 ± 1.2 [2–6]
TILsum maximum during the 8 first days in ICU	6.3 ± 2.6 [1–10]	18.9 ± 4.5 [11–28]

TILsum_High gathers the patients who have undergone at least one day with a TILsum equal or higher than 11 during their 8 first days in ICU. TILsum_Low gathers the other patients, without a day of extreme TIL.

#### Dataset 2

The second dataset contains 12 patients suffering a severe non penetrating TBI (GCS ≤ 8) admitted at the Universitary Hospital of Grenoble between August 2018 and April 2021 (RadioxyTC cohort; Health Data Hub index F20220207212747). TILsum score during the 5 first days in the ICU was retrieved and TILsum until the 8th day was estimated from clinical reports, in order to estimate the overall maximum TILsum score. This dataset was used to perform an external validation. Characterization of this dataset can be found in the Supplementary Table S4.

### Outcome

The TILsum score is computed daily from the list of interventions and treatments undergone by the patient in the ICU and is an integer between 0 and 38. Details about its computation can be found in the supplementary material S1. One can define a day of extreme management if the TILsum reaches 11 or more^19,20^. In this work, we tried to detect patients that underwent an extreme management day during the 8 first days in the ICU (group TILsum_High) from the others (group TILsum_Low).

### Preprocessing

All 84 CT-scans were extracted from the Hospital Storage System in DICOM and then converted to NIfTI format thanks to the MP3 software^21^. The brain was extracted with a MATLAB-based Skull removal algorithm^22^. Then, all images from a patient were rigidly co registered to his first CT-scan obtained at admission using the FLIRT algorithm from the FSL toolbox^23^ and finally resampled at 1mm^3^.

### Segmentations

On Dataset 1, we segmented TBI lesions on the CT-scans in 6 different ways. First, we applied the DeepMedic-based^24^ Convolutional Neural Network (CNN) called BLAST-CT^14^, which aims to automatically segment 4 lesions typical of TBI : IPH, EAH, Od and IVH leading to the *BLAST-CT segmentation*. Then, this segmentation was manually corrected by JAdB and AA, respectively anesthesiologist and neuroradiologist with 2 and 10 years of experience, using ITK-SNAP software^25^, to obtain the *4-class manual segmentation*. This manual segmentation was then refined by splitting EAH between subdural hemorrhage (SDH), epidural hemorrhage (EDH) and subarachnoïd hemorrhage (SAH), and by distinguishing petechiae (Pe) from IPH, leading to the *7-class manual segmentation*. Finally, we used two transfer learning techniques to refine the automatic segmentations of BLAST-CT: a Fine-Tuning approach^26^ in order to obtain an automatic 4-class segmentation named *CNN2* and a Transfer Learning approach^27^ which lead to two 7-class automatic segmentations named *CNN3* and *CNN4*, depending on their initialization. The principal characteristics of the 4 different automatic segmentations evaluated are summarized on Table 2. On Dataset 2, manual segmentations at 4 and 7 classes were drawn by FDH (anesthesiologist, 1 year of experience), JAdB, and AA.

**Table 2:** Description of the 4 CNN using to automatically segment TBI lesions on CT-scans.

Name of the model	Number of lesions segmented	Weights initialization	Trained on our data?	Training method
CNN1: BLAST-CT	4	BLAST-CT weights	No	Not applicable
CNN2	4	BLAST-CT weights	Yes	Fine tuning
CNN3	7	Random weights	Yes	Classical training
CNN4	7	CNN2 weights	Yes	Transfer learning

### Features extraction

A structural atlas was retrieved and adapted from the FSL toolbox^23^ and a CT-scan template was downloaded from^28^. The atlas and template were co-registered, first linearly thanks to the FLIRT algorithm^23^ and then elastically using ANTS^29^, to all CT-scans acquired at admission. All voxels inside the brain were thereby ascribed to one of the 11 areas of the atlas: Frontal (FL), Parietal (PL), Occipital (OL) and Temporal Lobes, but also Caudate, Cerebellum, Insula, Putamen, Thalamus, the rest of the brain, mainly composed of the ventricles, and the extra-cerebral space.

**Figure 2:**
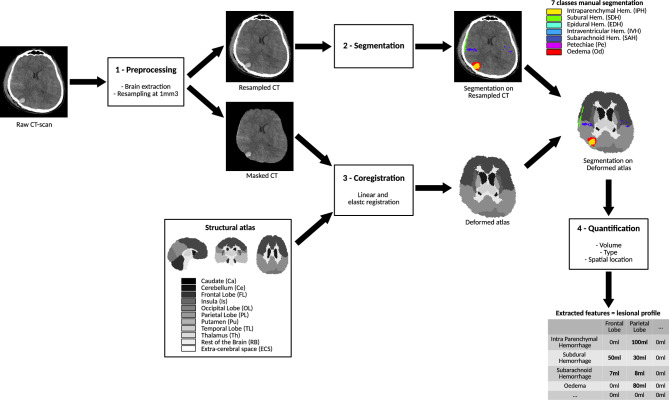
Overview of the lesion volume quantification by lesion type and spatial location.

For the 3 segmentation approaches (BLAST-CT/4-class manual/7-class manual), we extracted, as illustrated on Fig. 2, the volume of each injury (respectively 4/4/7) in each area of the atlas (11), leading to respectively 44/44/77 metrics. For each of these segmentations, we combined these metrics to perform 7 experiments, each with a different set of metrics, detailed on Table 3.

**Table 3:** Nature of input metrics for the 7 experiments.

Experiment number	Metrics	Number of metrics
Exp 1	Age, GCS, MAP, Hb, antiaggregants	5
Exp 2	Exp1 set + Marshall + Rotterdam scores	7
Exp 3	Global volume of lesion in the brain	1
Exp 4	Volume of lesion per type of injury (e.g., 300 mm^3^ of Intraparenchymal Hemorrhage, …)	4 for 4-class segmentations, 7 for 7-class segmentation
Exp 5	Volume of lesion per spatial location (e.g., 300 mm^3^ of lesion in the Frontal Lobe, …)	11
Exp 6	Exp4 set concatenated with the Exp5 set	15 for 4-class segmentations, 18 for 7-class segmentation
Exp 7	Volume of lesion per type of injury and spatial location (e.g., 300 mm^3^ of intraparenchymal Hemorrhage in the Frontal Lobe, …)	44 for 4-class segmentations, 77 for 7-class segmentation

### Classification

For each of the 7 experiments and each of the 3 segmentations (BLAST-CT/4-classes manual/7-class manual), we trained a classifier to predict whether patients belong to the TILsum_Low or the TILsum_high group. Our classifier was designed using the PhotonAI toolbox^30^, which proposes machine learning pipelines, containing data preprocessing, data augmentation, feature selection, hyperparameter optimization and model evaluations. Due to the small number of patients in our dataset, we used a state-of-the-art method: a nested cross-validation^31,32^, on the train and validation sub-datasets to assure statistical robustness in the tuning and evaluation of our classifiers. This procedure is detailed on Supplementary Figs. S1 and S2.

### Classification model selection

To compare our classification models, optimized and trained with different sets of metrics and different segmentations, we considered their global Area Under the Curve (AUC), of the Receiver-Operating Characteristic (ROC) curve, computed by the PhotonAI toolbox, and summarizing the sensibility and specificity of a binary classification model. As a direct implication of the classification procedure, each experiment resulted in 60 AUC values. To evaluate our models, we eventually considered the mean and standard deviation of these 60 AUC values. Significance of the difference of AUC distributions was assessed with non-parametric two-sided Mann–Whitney tests. After the training, we evaluated the importance of each feature of the best model, using the Mean Decrease impurity, in order to identify the key metrics that influence the model prediction.

### Validations

We performed 2 validations. First we used the test sub-dataset of Dataset1 (6 patients) to evaluate our segmentations and classification models on the same type of data as the ones used for the training. Then, we used Dataset2 (12 patients) to evaluate our algorithms on data from the same center but from another study. We evaluated the accuracy of segmentation and classification models on these 2 validation datasets as described below:

#### Segmentation evaluation

We evaluated the segmentations by computing the DICE score^12^, with the toolbox^33^, between the 4 automatic segmentations and the related manual segmentation, on each lesion but also on the overall segmentation, obtained by merging all the classes into a unique class representing the lesional tissues (All). We then separately compared the two 4-class CNN and the two 7-class ones. Significance of the difference of Dice scores distribution was assessed with non-parametric two-side Wilcoxon tests.

#### Classification evaluation

We retrieved the best classification model from the nested cross validation using 4-class segmentations and 7-class segmentations, leading to 2 classification models: “*4-class Classification model*” and “*7-class Classification model*”, both aimed at predicting our TILsum-based outcome. We then extracted the Volume/Type/Spatial location from the 6 different segmentations available (2 manuals and 4 automatics) and applied the related classification model. Finally, we measured and compared the accuracy of classification for each segmentation. Others evaluation metrics were measured and included in the supplementary material S1.

### Ethics approval and consent to participate

The study Radiomic-TBI involving human participants was reviewed and approved by the French institution Comité de protection des personnes (Radiomic-TBI cohort; NCT04058379, first posted: 15 august 2019). Informed consent was obtained from all subjects and/or their legal guardian(s). The study RadioxyTC was also allowed by the French Direction de la Recherche Clinique et de l’Innovation and registered on the Health Data Hub (Radioxy-TC cohort; Health Data Hub index F20220207212747, first posted: 7 February 2022). Patients were individually informed, but no written informed consent was required, although patients had the opportunity to decline their participation in the study. These studies were carried out in accordance with the french regulation.

## Results

### Cohort characterization

The cohort characterization can be found on Table 1, for both groups TILsum_High and TILsum_Low used for the classification task. One can observe the unbalanced distribution of men and women and of antiaggregants in the two groups. As expected, the imaging scores (Marshall and Rotterdam) are lower in the TILsum_Low group than the TILsum_High one, whereas GCS are higher. The characterization of the second split of Dataset1 (train, validation and test sub-datasets) is provided in the Supplementary Table S5.

### Classification model TILsum prediction

The mean and standard deviation of the AUC on the outer folds of the nested cross validation are shown on Table 4. The results show that clinical metrics fail to predict our TILsum based outcome. Adding manually estimated imaging scores improves the prediction power to 66 ± 24%. The global volume of injury does not show good predictions but splitting it by type or spatial location improves the prediction. The best model using 4-class segmentation is obtained for Exp7 and the 4-class manual segmentation (AUC = 74 ± 26%, Bias corrected and accelerated two-sided bootstrap 99-confidence interval [65, 83]). For the best model resulting from this nested cross-validation, called “*4-class Classification model*”, the most important metrics, regarding the Mean Decrease impurity, are the volume of EAH in FL (importance of 38%) and in OL (31%). The overall best result is obtained for Exp7 and the 7-class manual segmentation (AUC = 89 ± 17%, Bias corrected and accelerated two-sided bootstrap 99-confidence interval [83, 94]). For this best model, called “*7-class Classification model*”, the two most important metrics used for achieving the prediction are the volume of SDH in PL (importance of 47%) and in FL (33%). The second best model, obtained for type metrics (Exp4) and the 7-class manual segmentation (AUC = 75% ± 27%), relies the most on the volume of SDH (importance of 53%), SAH (26%), and IVH (20%). These results need to be seen through the prism of the small sample size.

**Table 4:** AUC (Mean ± STD) on the outer folds of the models trained for 3 different segmentations (1 automatic and 2 manual) and 7 metrics sets.

	Exp 1: clinical	Exp 2: clinical + imaging scores	Exp 3: volume total lesion	Exp 4: volume per type	Exp 5: volume per spatial location	Exp 6: concatenation volume per type and volume per spatial location	Exp 7: volume per type and spatial location
4 classes BLAST-CT segmentation	54 ± 24	66 ± 24	59 ± 24	61 ± − 27	65 ± 25	64 ± 25	60 ± 23
4 classes manual segmentation			63 ± 22	69 ± 24	71 ± 25	68 ± 26	74 ± 26
7 classes manual segmentation				75 ± 27		73 ± 24	**89 ± 17**

Results from the nested cross-validation procedure on data from the Train and Validation sub-datasets of Dataset1 (23 patients). Best result in bold.

Regarding the segmentations, the 7-class manual segmentation shows better results than the 4-class manual segmentation, which is itself better than BLAST-CT on all experiments. On the Exp7, according to the bilateral Mann–Whitney test, 7 classes manual segmentation performed significantly better than the 2 others segmentations (p < 0.01 compared to 4-class manual segmentation, p < 0.001 compared to BLAST-CT segmentation) and the 4 classes manual segmentation performed significantly better than the BLAST-CT segmentation (p < 0.01).

### Validations

#### Segmentation evaluation

##### Internal validation

**Figure 3:**
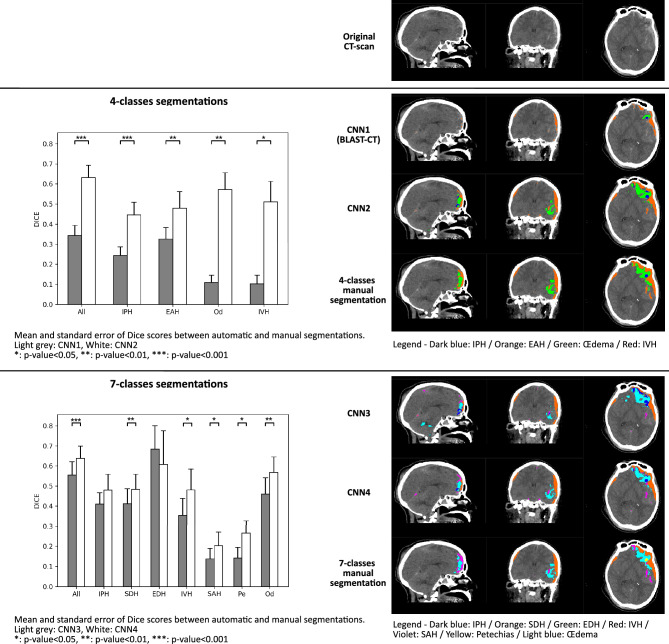
Barplots of the DICE scores (mean and standard error) computed on each lesion and on overall lesions between automatic and manual segmentations on the test sub-dataset of Dataset1 (6 patients—17 CT-scans). Upper part shows the comparison of CNN1 and CNN2, as well as an illustration of the resulting lesions. Lower part shows the comparison of CNN3 and CNN4, as well as an illustration of the resulting lesions. Significance was assessed with non-parametric two-side Wilcoxon tests. *p < 0.05.

The comparison of Dice scores between 4-class automatic segmentations (CNN1 and CNN2) and the 4-class manual segmentation is displayed on Fig. 3 and detailed on Supplementary Table S6, for each lesion type and for the overall lesion, as well as an illustration of the resulting segmentations.

On every lesion, CNN2 (average DICE score on overall lesions = 0.63), showed statistically significantly better results than CNN1 (BLAST-CT) (0.34). The gain is particularly large on Od and IVH lesions, where CNN1 performs poorly.

The comparison of Dice scores between 7-class automatic segmentations (CNN3 and CNN4) and the 7-class manual segmentation are displayed on Fig. 3 and detailed on Supplementary Table S7, for each lesion type and for the overall lesion, as well as an illustration of the resulting segmentations.

On the overall lesion, CNN4 (average DICE score on overall lesions = 0.64), showed statistically significantly better results than CNN3 (0.55). On the EDH lesion, results are unexpected, as CNN3 is better than CNN4, but not significatively, probably due to the small sample size of images containing this lesion.

##### External validation

**Figure 4:**
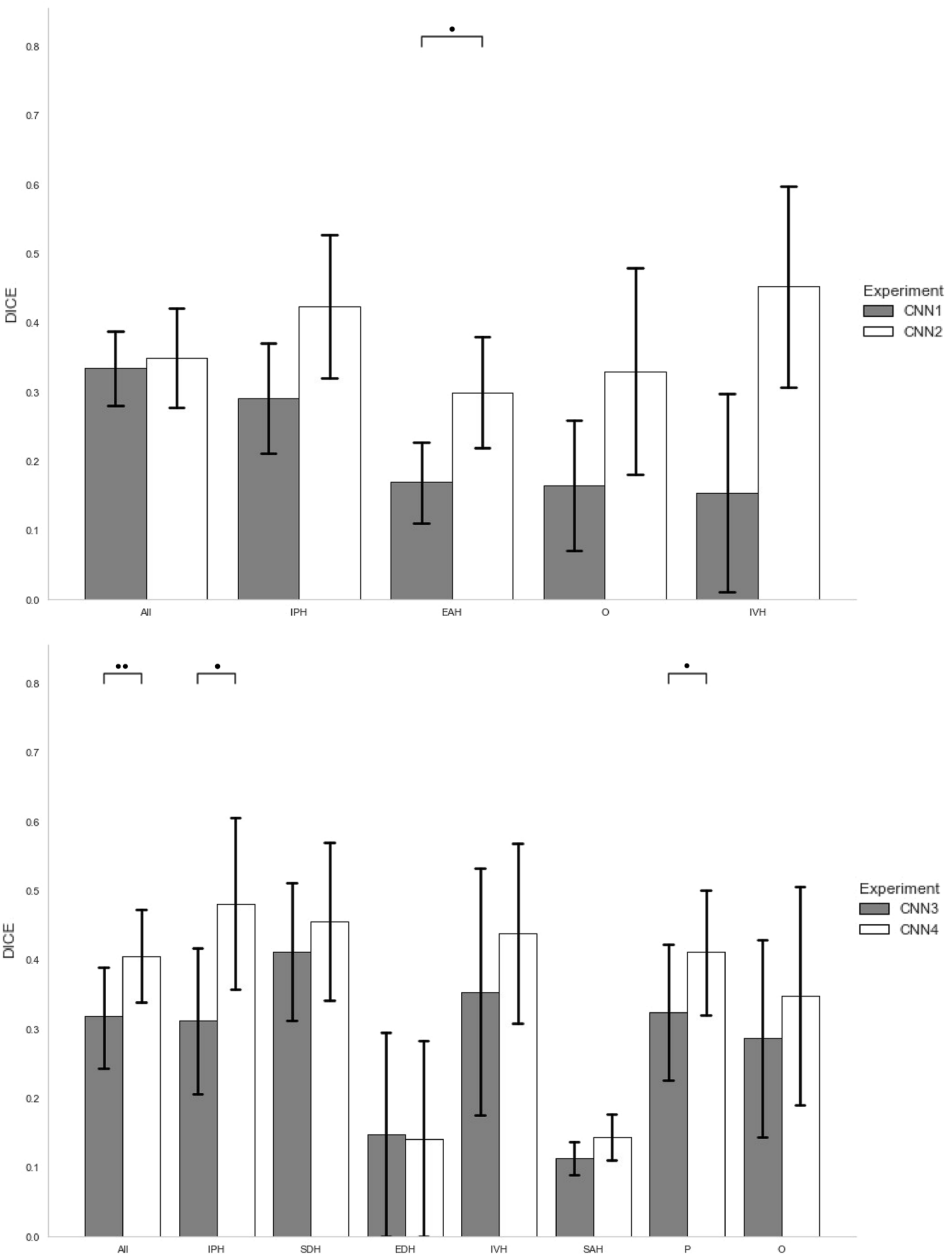
Barplots of the DICE scores (mean and standard error) computed on each lesion and on overall lesions between automatic and manual segmentations on Dataset2 (12 patients—12 CT-scans). Upper part shows the comparison of CNN1 (grey) and CNN2 (white). Lower part shows the comparison of CNN3 (grey) and CNN4 (white). Significance was assessed with non-parametric two-side Wilcoxon tests.*p < 0.05, **p-value < 0.01.

Comparison of the DICE scores computed on Dataset2 (12 patients) between segmentations resulting from CNN1 to CNN4 and manual segmentations are shown on Fig. 4 and detailed on Supplementary Tables S8 and S9.

#### Classification evaluation

Prediction accuracy of the 2 best classification models (“*4-class Classification model*” and “*7-class Classification model*”, see section “Classification model TILsum prediction”) on the test sub-dataset of Dataset1 (Internal validation—6 patients) and on the Dataset2 (External validation—12 patients) for 6 segmentations (BLAST-CT, our 3 automatic segmentations, and the 2 manual segmentations) are shown on Table 5.

**Table 5:** Accuracies of the classification on internal and external validation datasets, for 6 segmentations (4 automatic, 2 manual).

Classification model	Applied segmentation	Internal validation accuracy	External validation accuracy
4-class classification model	CNN1 (BLAST-CT)	50% (3/6)	67% (8/12)
	CNN2	67% (4/6)	67% (8/12)
	Manual4	67% (4/6)	67% (8/12)
7-class classification model	CNN3	83% (5/6)	75% (9/12)
	CNN4	83% (5/6)	83% (10/12)
	Manual7	83% (5/6)	67% (8/12)

Best accuracies were obtained for 7-class segmentations. CNN1 was only able to correctly classify 50% and 67% respectively internally and externally. The two transfer learning automatic segmentations were able to reach the same accuracy as manual segmentations internally, and with the 7-class segmentation, transfer learning automatic segmentation outperformed the external prediction made with the manual segmentation (10/12 vs 8/12).

## Discussion

In this study, we quantified the ability of volume, spatial location and type of brain lesions observed on admission CT-scans to predict the therapeutic intensity level of TBI patients within the first week in ICU. Volumes of 7 different lesions on 11 structural zones were able to predict this outcome with a mean AUC of 89% and a standard deviation of 17%. Although the small sample size limits the conclusions of this work, the most influential metrics are the volume of lesions located in brain lobes, and especially the volume of subdural hemorrhage. This result is coherent with the medical experience about the large impact of SDH, often located in the frontal and parietal lobes, on medical care^34^, and with the study pre-published by Rosnati et al. in which 6-months mortality of TBI patients has been predicted from frontal EAH lesions^11^. This recent study was conducted on more than 600 patients but only considered the 4-class BLAST-CT segmentation, when our study, conducted on way less patients, predicted a short-term outcome and exploited 7-class segmentations to highlight the influence of SDH.

We also highlighted the low predictive power of BLAST-CT to automatically predict our TILsum-based outcome. This lack of prediction could be explained by the poor segmentation obtained using BLAST-CT on our brain CT-scans. Indeed, although BLAST-CT was developed on large multicentric datasets (n = 839) the DICE obtained using BLAST-CT on our patients were lower than those published by Monteiro et al.^14^. In order to improve this automatic segmentation, we used 2 transfer learning approaches. First, we showed on the test sub-dataset of Dataset1 that the fine tuning of a deep learning algorithm on a small local dataset (n = 67 for training, consisting in the merge of train and validation sub-datasets) leads to a significantly increased segmentation accuracy. This result can change the classical paradigm in which the objective of segmentation studies is to train an algorithm on a large multicentric dataset to learn and overcome the intersite variability. It might then be possible to easily fine-tune with a few images an already trained algorithm in order to learn the specificities of a study. The second approach, aimed at automatically segmenting 7 lesions from the 4-class segmentation algorithm by transfer learning, showed good results on highly represented lesions but was less accurate on poorly represented ones (such as petechiaes or EDH), a classical behavior in machine learning.

Finally, in order to link our segmentation work to a clinically relevant issue, we validated our results by using the improved segmentations to predict our clinical outcome on the test sub-dataset of Dataset 1. We showed that our improved segmentations predict the TILsum based criteria as the manual ones do. Segmentation and classification were then validated on a new external dataset (Dataset2) leading, as obtained on the internal validation, to better results with transfer learning approaches, and a prediction accuracy of 83% with automatic segmentation (10/12), better than the one obtained with manual segmentation (8/12), which is counterintuivite. This former result could be explained by the unperfect manual segmentation, as illustrated on the Supplementary Fig. S3. In this case, segmenting using deep learning would undoubtedly have produced a more accurate segmentation.

Compared to recent automated hemorrhage segmentation literature, most of the studies do not discriminate SDH from others hemorrhage. While Yao et al. only focused on hematoma volume estimation, Monteiro et al. merged SDH with SAH and EDH. To our knowledge, the study of Farzaneh et al. is the only study to segment SDH, which is crucial to discriminate against short term evolution, as shown by our classification study. Farzaneh et al. study reached a Dice score of more than 0.75 by combining deep learning and classical image processing methods, outperforming our external validation SDH Dice score of 0.46. While differences in patients' inclusion and statistical evaluation methods might explain part of this score, it is probable that non deep learning post processing might improve deep learning segmentation by making them closer to neuroradiologists segmentations.

To our knowledge, we developed the first automatic tool to predict the intensity level of medical care from CT-scans of brain-injured patients, linking image processing to clinical care. In order to share our CT-scan quantification tool described on Fig. 2 and named CT-TIQUA v1.4, we encapsulated our best segmentation model, atlas registration, and volumes extraction in a docker container and integrated it on the computing platform VIP^36^, that enables anyone to execute any pipeline on dedicated computing resources from the web-interface: https://vip.creatis.insa-lyon.fr/. This tool is the first to provide a 7-class segmentation of TBI injuries as well as registered atlas. Its universal utilization might allow to easily try it on another study or task.

Of course, this study has some limitations. First, our datasets are small, leading to unstable classification performances and all these results must be validated on larger and multicentric cohorts before any further use in clinical practice. Secondly, since this is the first study to predict the therapeutic intensity level, we cannot compare ourselves to the literature and evaluate the quality of our CT-scans quantification. To overcome this limitation, one will soon evaluate the prediction of the 6-months mortality to be able to compare our classification results with the ones of the most similar study conducted by Rosnati et al.^11^.

To conclude, we believe that the automatic quantification of CT-scans to predict short-term outcome of TBI patients has the potential to bring reproducible and reliable information that can help improve clinical care. One must multiply the research studies in this way but also investigate the lesions evolution on repeated CT-scans, that might contain crucial information currently unused.

### Supplementary Information

Supplementary Information.

## Data Availability

The datasets analysed during the current study are available from the corresponding author on reasonable request.

## Abbreviations

CT: Computed Tomography
AI: Artificial Intelligence
TBI: Traumatic brain injury
TILsum: Therapeutic intensity level summary
ICU: Intensive care unit
CNN: Convolutional neural network
AUC: Area under the curve
