# Supplementary material for: Prediction of therapeutic intensity level from automatic multiclass segmentation of traumatic brain injury lesions on CT-scans
Source: Sci Rep. 2023 Nov 17;13:20155. doi: 10.1038/s41598-023-46945-9 (PMC10656472; doi:10.1038/s41598-023-46945-9)
Supplement: Supplementary file 1 — Supplementary Information. [file 41598_2023_46945_MOESM1_ESM.docx]

**Supplementary material**

***Classification procedure***

Classification task was performed using the PHOTONAI framework [^1^](https://www.zotero.org/google-docs/?9CrmZD). A nested cross validation was used with as outer loop a Repeated Stratified K-fold cross validation (10 times repeated, K=6) and as inner loop a Repeated Stratified K-fold cross validation (10 times repeated, K=5). The final AUC used to evaluate a set of metrics in our outcome prediction is the mean AUC on the 60 AUC values resulting from the outer loop. Detailed process on supplementary_figure 1.

**Supplementary_figure 1:** nested cross validation process used in this study.

Each training followed the same process, detailed on supplementary_figure 2. First, all metrics were scaled between 0 and 1. Then, since the repartition of our 29 patients between TILsum_High and TILsum_Low was 20/9 (16/7 in the concatenation of the training and validation subdatasets), we randomly selected samples to finally have a 50/50% repartition between the 2 groups. We then only selected 4 metrics among the input metrics thanks to the SelectKBest method and the f_classif score function. Shortly, it computes the ANOVA F-value between each of the input metrics and the output (our outcome) and keeps the 4 metrics corresponding to the highest scores. These selected metrics finally feed the Random Forest classifier. We tested several values for the classifier’s hyperparameters, leading to 90 different possible settings, each evaluated in the nested cross-validation process. On the validation dataset, the minmax scaler and the selection of the 4 formerly selected metrics were applied before the application of the model trained on the train dataset. All initialization seeds were set to 42 to obtain non-stochastic results.

**Supplementary_figure 2**: Preprocessing and classification process.

***Scanner models and acquisition parameters***

CT-scans were acquired with a GE Healthcare Revolution GSI scanner. Acquisition parameters were: Voltage of 120kV, collimation of 20mm (32 x 0.625), intensity modulation (noise index equal to 6.5, intensity between 80mA and 250mA), helical pitch of 0.53, rotation time equal to 0.8s, 1.25mm slice reconstruction with ASIR50 iterative algorithm. For this acquisition protocol, median Computed Tomography Dose Index (CTDI) is equal to 35mGy, and median Dose Length Product (DLP) is equal to 630mGy.cm. For comparison, in France, reference levels for this examination are equal to 46mGy for CTDI and 850mGy.cm.These values are associated with 1 CT-scan. Patients included in Dataset 1 undergo 2 or 3 examinations. Some of these patients did not undergo a third CT-scan because of transport difficulties due to critical clinical state.

***Additionnal experiments***

We conducted 6 other experiments, similar to experiments in the main manuscript but with clinical data included as input metrics. Supplementary_Table 1 details the 6 experiments and Supplementary_Table 2 gathers the results.

| **Experiment number** | **Metrics** | **Number of metrics** |
| --- | --- | --- |
| Exp 3_b | Metrics from Exp 3 + metrics from Exp 1 | 6 |
| Exp 4_b | Metrics from Exp 4 + metrics from Exp 1 | 9 for 4-class segmentations,  12 for 7-class segmentation |
| Exp 5_b | Metrics from Exp 5 + metrics from Exp 1 | 16 |
| Exp 6_b | Metrics from Exp 6 + metrics from Exp 1 | 20 for 4-class segmentations,  23 for 7-class segmentation |
| Exp 7_b | Metrics from Exp 7 + metrics from Exp 1 | 49 for 4-class segmentations,  82 for 7-class segmentation |
| Exp 8 | Metrics from Exp 7 + metrics from Exp 2 | 51 for 4-class segmentations,  84 for 7-class segmentation |

**Supplementary_Table 1** : Nature of inputs metrics for 5 additional experiments

|  | Exp 3_b | Exp 4_b | Exp 5_b | Exp 6_b | Exp 7_b | Exp8 |
| --- | --- | --- | --- | --- | --- | --- |
| 4 classes BLAST-CT segmentation | 52 ±27 | 56 ±-29 | 66 ±25 | 61 ±27 | 65 ±25 | 71 ±25 |
| 4 classes manual segmentation | 54 ±27 | 63 ±26 | 71 ±21 | 68 ±26 | 73 ±25 | 67 ±28 |
| 7 classes manual segmentation |  | 70 ±25 |  | 75 ±23 | **86 ±18** | 83 ±20 |

**Supplementary_Table 2:** AUC (Mean±STD) on the outer folds of the models trained for 3 different segmentations (1 automatic and 2 manual) and 6 additional metrics sets. Results from the nested cross-validation procedure on data from the Train and Validation sub-datasets of Dataset1 (23 patients). Best result in bold.

***Details about internal and external classification validation performances***

Supplementary_Table 3 gathers supplementary evaluation metrics that describe our internal and external validation.

Additional metrics: balanced accuracy (bAcc), Specificity (Sp), Sensitivity (Se), Precision, F1 score, and Area Under the Receiver Operating Curve (AUC)

| Classification model | Applied segmentation | Internal Validation | | | | | | External Validation | | | | | |
| --- | --- | --- | --- | --- | --- | --- | --- | --- | --- | --- | --- | --- | --- |
|  |  | bAcc  (%) | Sp  (%) | Se  (%) | Precision  (%) | F1 score  (%) | AUC  (%) | bAcc  (%) | Sp  (%) | Se  (%) | Precision  (%) | F1 score  (%) | AUC  (%) |
| 4-class Classification model | CNN1  (BLAST-CT) | 38 | 75 | 0 | 0 | 0 | 50 | 60 | 100 | 20 | 100 | 33 | 74 |
|  | CNN2 | 75 | 50 | 100 | 50 | 67 | 88 | 63 | 86 | 40 | 67 | 50 | 51 |
|  | Manual4 | 63 | 75 | 50 | 50 | 50 | 81 | 60 | 100 | 20 | 100 | 33 | 74 |
| 7-class Classification model | CNN3 | 88 | 75 | 100 | 67 | 80 | 94 | 70 | 100 | 40 | 100 | 57 | 70 |
|  | CNN4 | 88 | 75 | 100 | 67 | 80 | 94 | 80 | 100 | 60 | 100 | 75 | 74 |
|  | Manual7 | 88 | 75 | 100 | 67 | 80 | 75 | 60 | 100 | 20 | 100 | 33 | 74 |

**Supplementary_Table 3:** Evaluation metrics of classification models on internal and external validation datasets, for 6 segmentations (4 automatic, 2 manual). Metrics include balanced accuracy (bAcc), Specificity (Sp), Sensitivity (Se), Precision, F1 score, and Area Under the Receiver Operating Curve (AUC)

Supplementary_Figure 3 shows on a patient from Dataset 2, who was misclassified by our classification model when using the manual segmentation but well classified with the automatic one, the segmentation of subdural hemorrhage (SDH) obtained with the manual segmentation and the automatic segmentation from CNN 4. The arrows point to a difference in the SDH segmentation in the frontal lobe, which is a key metric for the success of the TILsum prediction and therefore could explain the misclassification.

**Supplementary_Figure 3**: Comparison between segmentation of SDH between automatic segmentation (middle row) by CNN4 and manual segmentation (bottom row), on one of the two patients misclassified when using the manual segmentation but well classified with the automatic one. The arrows point to a difference in the SDH segmentation in the frontal lobe, which is a key metric for the success of the TILsum prediction.

***Characterization of the Dataset 2***

Supplementary_Table 4 shows the Characterization of the Dataset 2 cohort.

|  | **TILsum_Low** | **TILsum_High** |
| --- | --- | --- |
| **Nb Patients** | 7 | 5 |
| **Age (years)** | 32,3 +-14,4 [19-55] | 44,6 +-12,7 [27-60] |
| **Sex** | 7M / 0F | 3M / 2F |
| **Weight (kg)** | 76,1 +-7,0 [70-90] | 86,0 +-14,9 [72-110] |
| **Height (cm)** | 175,-7 +-4,5 [170-180] | 174,0 +-9,6 [165-190] |
| **GCS** | 5,4 +-1,8 [3-7] | 6,6 +-0,5 [6-7] |
| **TILsum maximum estimated during the 8 first days in ICU** | 6,6 +-2,4 [4-10] | 13,0 +-3,5 [11-19] |

**Supplementary_Table 4 :** Characterization of the Dataset 2 cohort. (Mean +-STD [Min-Max]). TILsum_High gathers the patients who have undergone at least one day with an estimated TILsum equal or higher than 11 during their 8 first days in ICU. TILsum_Low gathers the other patients, without a day of extreme therapeutic intensity level.

***Segmentation procedure***

The Dataset1 was splitted in train/validation/test sub-datasets in order to get balanced groups in terms of TILsum_Low/TILsum_High repartition but also in terms of lesion volume, for each lesion type.

Supplementary_Table 5 characterizes these 3 sub-datasets.

|  | Train sub-dataset | Validation sub-dataset | Test sub-dataset |
| --- | --- | --- | --- |
| Number of patients | 17 | 6 | 6 |
| TILsum_Low / TILsum_High | 12/5 | 4/2 | 4/2 |
| Number of CT-scans | 50 | 17 | 17 |
| Volume total of IPH (cm3) | 475,6 (68%) (36) | 109,1 (16%) (14) | 109,9 (16%) (12) |
| Volume total of SDH (cm3) | 810,0 (61%) (39) | 229,5 (17%) (14) | 282,7 (21%) (13) |
| Volume total of EDH (cm3) | 235,1 (65%) (15) | 66,2 (18%) (1) | 59,6 (17%) (3) |
| Volume total of IVH (cm3) | 12,2 (64%) (17) | 3,3 (17%) (7) | 3,7 (19%) (6) |
| Volume total of SAH (cm3) | 211,5 (68%) (34) | 49,9 (16%) (17) | 48,0 (16%) (12) |
| Volume total of Petechiae (cm3) | 5,6 (62%) (15) | 1,9 (20%) (8) | 1,6 (18%) (11) |
| Volume total of Oedema (cm3) | 1177,4 (62%) (37) | 334,2 (18%) (13) | 373,9 (20%) (11) |
| Total volume of lesion (cm3) | 2927,6 (64%) (50) | 794,2 (17%) (17) | 879,4 (19%) (17) |

**Supplementary_Table 5** : Characterization of training, validation and test sub-datasets used for the training and evaluation of the segmentation experiments (volume of lesion (Proportion) (Number of CT-scans with presence of lesion)

***Deep learning Trainings***

We used the MONAI framework (<https://github.com/Project-MONAI/MONAI>), based on the PyTorch toolbox (<https://pytorch.org/>) . All the trainings were trained on 1500 epochs, with a batch size of 2 and a DICE/Cross Entropy loss function (<https://docs.monai.io/en/stable/losses.html#diceceloss>). As a stopping criteria, we stopped the training if the validation loss did not improve in 200 validations, where one validation happened every 2 epochs. The selected model was the one that showed the best DICE score on the validation sub-dataset. The DeepMedic architecture takes as input a 110x110x110 patch and outputs a 30x30x30 segmentation. For the training, for each input CT-scan, we used 20 patches at every epoch and 40 for the validation, with a fixed probability to be centered on a lesion. Preprocessing and data augmentation was set as BLAST-CT [^2^](https://www.zotero.org/google-docs/?7SgIVW), except the random elastic deformation, that we did not implement. BLAST-CT algorithm gathers the outputs of 12 models trained on the same data but with a different random seed in order to limit stochasticity. Final segmentations were obtained by averaging the probabilities of belonging to each class of the 12 models. Then each voxel was attributed to the class with the highest probability. For evaluation, we only considered DICE scores on scans where the ground truth contained a lesion. If ground truth contained a lesion but the automatic segmentation did not retrieve a lesion, DICE score was equal to 0. All CNN models were trained on the cluster of the meso-center GRICAD, located in the University Grenoble Alpes, on NVIDIA Tesla V100 GPU devices.

The details of the different segmentation models evaluated in this study are given below:

CNN 1 : We applied BLAST-CT on our images without any re-training.

CNN 2 : Thanks to the same architecture, we re-trained BLAST-CT models to better segment the 4 same injuries as BLAST-CT. The former method to re-train a model is known as fine-tuning [^3,4^](https://www.zotero.org/google-docs/?dVMhHt). It allows to retrain a model already trained on other data by initializing the weights of the model to train to the ones of the former trained model. We therefore fine-tuned each of the 12 BLAST-CT models available here (<https://github.com/biomedia-mira/blast-ct/tree/master/blast_ct/data/saved_models>) on our 4-class manual segmentation, resulting in a segmentation named CNN2.

CNN 3 : We adapted the DeepMedic architecture to output 7 class, then we trained 12 models with different random seeds initialized with random weights, resulting in a segmentation named CNN3.

CNN 4 : We adapted the DeepMedic architecture to output 7 class, then we trained 12 models initialized with CNN2 weights when the layers of the 4-class and the 7-class architecture were of the same size, randomly initialized otherwise, resulting in a segmentation named CNN4. The former method is known as a Transfer Learning approach [^5,6^](https://www.zotero.org/google-docs/?loUUcb).

***TILsum computation***

The outcome we predicted is based on the TILsum score, computed daily following the table retrieved from the Common Elements section of the TBI-impact website : <http://www.tbi-impact.org/cde/mod_templates/T_TIL.9.1.pdf>

As analogy with the classical TIL score, one can define the level of the management care in basic/mild/moderate/extreme if it is higher than or equal to, respectively, 0/4/8/11.

This TILsum score was daily computed during the 8 first days of a patient in the ICU. The maximum TILsum of a patient during these 8 days was retrieved as the TILsum_max. We then separated patients into 2 groups: patients that underwent an extreme management day (TILsum_max ≥ 11) and patients that did not (TILsum_max < 11).

***Internal segmentation validation: DICE scores***

Supplementary_Table 6 gathers the Dice scores computed between automatic 4-class segmentations (CNN1 and CNN2) and manual ones during the internal validation.

|  | All | IPH | EAH | Od | IVH |
| --- | --- | --- | --- | --- | --- |
| CNN1 | 0.34 (0.05) | 0.24 (0.04) | 0.33 (0.06) | 0.11 (0.04) | 0.10 (0.04) |
| CNN2 | 0.63 (0.07) | 0.45 (0.06) | 0.48 (0.08) | 0.57 (0.08) | 0.51 (0.10) |

**Supplementary_Table 6**: Mean and standard error of the dice scores computed between automatic 4-class segmentations and manual ones during the internal validation.

Supplementary_Table 7 gathers the Dice scores computed between automatic 7-class segmentations (CNN3 and CNN4) and manual ones during the internal validation.

|  | All | IPH | SDH | EDH | IVH | SAH | Pe | Od |
| --- | --- | --- | --- | --- | --- | --- | --- | --- |
| CNN3 | 0.55 (0.07) | 0.41 (0.06) | 0.41 (0.08) | 0.68 (0.12) | 0.35 (0.09) | 0.14 (0.05) | 0.14 (0.05) | 0.46 (0.08) |
| CNN4 | 0.64 (0.06) | 0.48 (0.08) | 0.48 (0.07) | 0.61 (0.17) | 0.48 (0.10) | 0.20 (0.07) | 0.26 (0.06) | 0.57 (0.08) |

**Supplementary_Table 7**: Mean and standard error of the dice scores computed between automatic 7-class segmentations and manual ones during the internal validation.

***External segmentation validation: DICE scores***

Supplementary_Table 8 gathers the Dice scores computed between automatic 4-class segmentations (CNN1 and CNN2) and manual ones during the external validation.

|  | All | IPH | EAH | Od | IVH |
| --- | --- | --- | --- | --- | --- |
| CNN1 | 0.33 (0.06) | 0.29 (0.08) | 0.17 (0.06) | 0.16 (0.10) | 0.15 (0.15) |
| CNN2 | 0.35 (0.07) | 0.42 (0.11) | 0.30 (0.08) | 0.33 (0.17) | 0.45 (0.22) |

**Supplementary_Table 8**: Mean and standard error of the dice scores computed between automatic 4-class segmentations and manual ones during the external validation.

Supplementary_Table 9 gathers the Dice scores computed between automatic 7-class segmentations (CNN3 and CNN4) and manual ones during the external validation.

|  | All | IPH | SDH | EDH | IVH | SAH | Pe | Od |
| --- | --- | --- | --- | --- | --- | --- | --- | --- |
| CNN3 | 0.32 (0.08) | 0.31 (0.12) | 0.41 (0.11) | 0.15 (0.15) | 0.35 (0.22) | 0.11 (0.03) | 0.32 (0.11) | 0.29 (0.15) |
| CNN4 | 0.41 (0.07) | 0.48 (0.13) | 0.46 (0.12) | 0.14 (0.14) | 0.44 (0.22) | 0.14 (0.04) | 0.41 (0.10) | 0.35 (0.17) |

**Supplementary_Table 9**: Mean and standard error of the dice scores computed between automatic 7-class segmentations and manual ones during the external validation.

***ATLAS modification***

The template was retrieved from [^7^](https://www.zotero.org/google-docs/?0hI93m). The initial atlas was retrieved from FSL [^8^](https://www.zotero.org/google-docs/?4k4YQs) (/fsl/data/atlases/MNI/MNI-maxprob-thr0-1 mm.nii.gz) and was modified as follow:

- The center of the brain was defined as a new class (Rest of the brain (RB)). This class was manually added by filling the central void with ITK-SNAP software.
- The atlas was manually registered to the template with FSLeyes software (translation, rotation, scaling).

***Number of samples from Dataset 1***

Dataset 1 was initially acquired to estimate the correlation between the TILsum score and the volume of new lesions on CT-scans between the admission (D0) and the next day (D1). In order to highlight a correlation of 0.8, with a risk of 5% and a power of 80%, the number of 330 patients was computed on the software Stata ® version 15 (Stata Corporation 4905 Lakeway Drive College Station, TX 77845 USA).

**Supplementary references**

[1. Leenings R, Winter NR, Plagwitz L, Holstein V, Ernsting J, Sarink K, et al. PHOTONAI—A Python API for rapid machine learning model development. PLOS ONE. 21 juill 2021;16(7):e0254062.](https://www.zotero.org/google-docs/?zHgKTL)

[2. Monteiro M, Newcombe VFJ, Mathieu F, Adatia K, Kamnitsas K, Ferrante E, et al. Multiclass semantic segmentation and quantification of traumatic brain injury lesions on head CT using deep learning: an algorithm development and multicentre validation study. Lancet Digit Health. juin 2020;2(6):e314‑22.](https://www.zotero.org/google-docs/?zHgKTL)

[3. Tajbakhsh N, Shin JY, Gurudu SR, Hurst RT, Kendall CB, Gotway MB, et al. Convolutional Neural Networks for Medical Image Analysis: Full Training or Fine Tuning? IEEE Trans Med Imaging. mai 2016;35(5):1299‑312.](https://www.zotero.org/google-docs/?zHgKTL)

[4. Takahashi S, Takahashi M, Kinoshita M, Miyake M, Kawaguchi R, Shinojima N, et al. Fine-Tuning Approach for Segmentation of Gliomas in Brain Magnetic Resonance Images with a Machine Learning Method to Normalize Image Differences among Facilities. Cancers. janv 2021;13(6):1415.](https://www.zotero.org/google-docs/?zHgKTL)

[5. Cheplygina V, de Bruijne M, Pluim JPW. Not-so-supervised: A survey of semi-supervised, multi-instance, and transfer learning in medical image analysis. Med Image Anal. 1 mai 2019;54:280‑96.](https://www.zotero.org/google-docs/?zHgKTL)

[6. Morid MA, Borjali A, Del Fiol G. A scoping review of transfer learning research on medical image analysis using ImageNet. Comput Biol Med. 1 janv 2021;128:104115.](https://www.zotero.org/google-docs/?zHgKTL)

[7. Rajashekar D, Wilms M, MacDonald ME, Ehrhardt J, Mouches P, Frayne R, et al. High-resolution T2-FLAIR and non-contrast CT brain atlas of the elderly. Sci Data. 17 févr 2020;7(1):56.](https://www.zotero.org/google-docs/?zHgKTL)

[8. Jenkinson M, Beckmann CF, Behrens TEJ, Woolrich MW, Smith SM. FSL. NeuroImage. 15 août 2012;62(2):782‑90.](https://www.zotero.org/google-docs/?zHgKTL)
